# Spatial spillover and COVID-19 spread in the U.S.

**DOI:** 10.1186/s12889-021-11809-2

**Published:** 2021-09-27

**Authors:** John Ulimwengu, Aziza Kibonge

**Affiliations:** 1 International Food Policy Research Institute (IFPRI), Eye Street, 1201 I St NW, Washington, DC, 20005 USA; 2 Institution: Ohio University, 1 Ohio University, Athens, OH 45701 USA

**Keywords:** COVID-19, Vulnerability index, Spatial spillovers, Minorities, Pre-existing conditions, Transportation, Housing, Healthcare

## Abstract

**Background:**

This research estimates the effects of vulnerability on the spread of COVID-19 cases across U.S. counties. Vulnerability factors (Socioeconomic Status, Minority Status & Language, Housing type, Transportation, Household Composition & Disability, Epidemiological Factors, Healthcare system Factors, High-risk Environments, and Population density) do not only influence an individual’s likelihood of getting infected but also influence the likelihood of his/her neighbors getting infected. Thus, spatial interactions occurring among individuals are likely to lead to spillover effects which may cause further virus transmission.

**Methods:**

This research uses the COVID-19 community index (CCVI), which defines communities likely vulnerable to the impact of the pandemic and captures the multi-dimensionality of vulnerability. The spatial Durbin model was used to estimate the spillover effects of vulnerability to COVID-19 in U.S. counties, from May 1 to December 15, 2020.

**Results:**

The findings confirm the existence of spatial spillover effects; with indirect effects (from neighboring counties) dominating the direct effects (from county-own vulnerability level). This not only validates social distancing as a strategy to contain the spread of the pandemic but also calls for comprehensive and coordinated approach to fight its effects. By keeping vulnerability factors constant but varying the number of reported infected cases every 2 weeks, we found that marginal effects of vulnerability vary significantly across counties. This might be the reflection of both the changing intensity of the pandemic itself but also the lack of consistency in the measures implemented to combat it.

**Conclusion:**

Overall, the results indicate that high vulnerability in Minority, Epidemiological factors, Healthcare System Factors, and High-Risk Environments in each county and adjacent counties leads to an increase in COVID-19 confirmed cases.

**Supplementary Information:**

The online version contains supplementary material available at 10.1186/s12889-021-11809-2.

## Background

As of May 4, 2021, the Centers for Disease Control and Prevention (CDC) reported effects of the COVID-19 pandemic included 32,267,958 cases, and 574,679 deaths in the U.S. [1]. At the same time, there is increasing evidence that rates of infections and mortality from COVID-19 are not evenly distributed across locations and socio-economic groups. For example, the percentage of hospitalizations, intensive care unit admissions, and deaths were highest among persons aged 70 years or higher, regardless of underlying conditions [2]. Moreover, the presence of underlying health conditions increases vulnerability to severe complications from COVID-19 [3]. Furthermore, communities with high levels of poverty, high concentration of less-educated adults, and minorities are more likely to face devastating effects of the virus [4, 5]. As evidence suggests, lower socioeconomic status, homelessness, poverty, racial and ethnic disparities [6], and health-related factors [7, 8] may significantly contribute to the COVID-19 transmission and incidence rates, thereby accounting for some of the spatial variation.

These factors do not only influence an individual’s likelihood of getting infected but do also influence the likelihood of his/her neighbors getting infected. Spatial interactions occurring among individuals are likely to lead to spillover effects which may cause further virus transmission. Hence, analyzing the spread of COVID-19 without accounting for spatial disparities and spillover effects will lead to biased estimates and erroneous policy recommendations. This study aims at estimating the effects of vulnerability factors on COVID-19 cases while explicitly accounting for spatial spillover effects.

Although several studies have looked at disparities, vulnerability, and COVID-19 spread [9–13], none has examined the related spillover effects. This is particularly important as people travel across counties and states. McLaren (2020) examined the relationship between COVID-19 severity and racial composition and found that minority’s population share is strongly correlated with overall COVID-19 deaths [14]; neighborhoods that are predominantly comprised of Black and Hispanic persons are disproportionately at risk to COVID-19 infections and mortality [15]. Unemployment has also been a contributing factor as the share of people not working and thus not commuting is correlated with higher death rates [16]. Likewise, age structure matters as the proportion of people living in nursing homes were shown to be significant and persistent predictors of number of deaths [17].

Several indices have been developed to measure vulnerability and identify populations at risk of contracting the virus. The CDC’s Social Vulnerability Index (SVI) in particular, is widely used in health research and was recently applied to COVID-19 analysis in U.S. counties ([9, 11, 18]). Moreover, spatial models have been used to capture the dynamics of COVID-19 prevalence in U.S. counties as they partially explain the geographic disparities in COVID-19 period prevalence [19]. Indeed, substantial spatial heterogeneity is observed across U.S. counties, and the association between case counts of COVID-19 infection and social vulnerability in U.S. counties allowed the most vulnerable counties to be identified. These counties had on average higher incidence rates compared to least vulnerable counties [9, 20, 21]. Neelon et al. (2021) reported that COVID-19 disproportionately affected less vulnerable counties at the outset of the pandemic, before spreading to more vulnerable counties over time [11].

## Methods

### Social interactions models

Social interaction models study how interaction among individuals or entities can lead to collective behavior and aggregate patterns [22]. These models incorporate endogenous effects, contextual effects, and unobserved correlation effects. As pointed out by Lewnard et al. (2020) in the health literature, symptoms of COVID-19 vary – some infected people seem to experience no symptoms, while others suffer from many symptoms which typically take from two to fourteen days to appear after infection [23]. The rate at which a susceptible person in a given subpopulation acquires the infection is determined by his/her interactions with infectious persons, either in the home subpopulation or in its neighboring subpopulations [18].

**Fig. 1:**
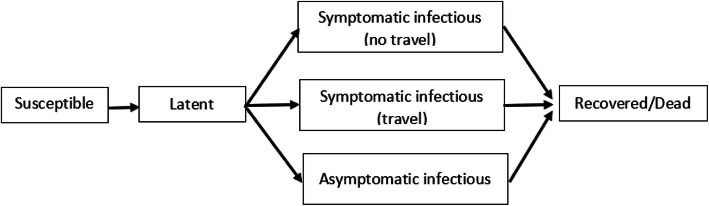
Compartmental structure of the epidemic model within each subpopulation, adapted from [18]

In general, latent persons will transit to asymptomatic infectious stage with high probability of becoming symptomatic infectious (Fig. 1). Infectious persons with symptoms can further be divided between those who can travel and those who are travel restricted. This paper focuses on those who can transmit the disease through social interactions, which further sparks disease spread across locations.

**Fig. 2:**
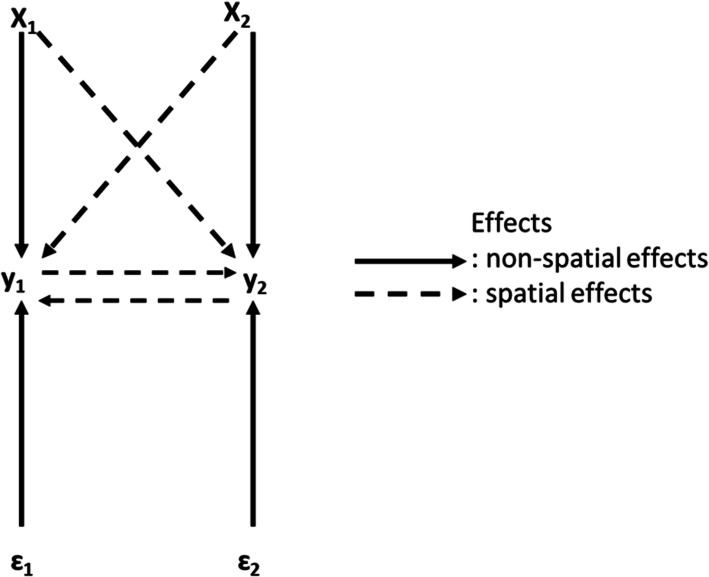
SMD for 2 locations

Infections occur mainly though exposure to respiratory droplets when a person is in close contact with someone who has COVID-19 (six feet of one another) [24]. Thus, there is potential for spillover effects because people who are physically near a person with COVID-19 or have direct contact with that person are at greater risk of infection [25]. Qu et al. (2012) argue that ignoring the fact that spatial or social interactions among individuals simultaneously determine the outcomes can cause biased estimates for the effects of exogenous variables and may also underestimate their influences [26]. Indeed, location *i* ’s exogenous attributes will not only influence its own rate of pandemic spread but, due to spatial interactions, its neighbors’ pandemic rate may also be influenced, which will in turn affect location *i* ’s pandemic level and so on as described in Fig. 2.

Given the presence of spatial interactions, the number of infections (*y*) and its determinants (*X*), we implement a spatial Durbin model (SDM) of the following form [27]:

1 $$ y=\rho Wy+ X\beta + WX\gamma +\varepsilon $$

where *y* is an n × 1 vector of observations on the dependent variable, *X* is an n × K matrix of observations on the explanatory variables, *ρ* is n × 1 vector of spillover parameter, *β* and *γ* are K × 1 vectors of parameters, *W* is n × n spatial weight matrix that capture the structure spatial interactions, and *ε*~*N*(0, *I*_*n*_) is n × 1 vector of the error term.

Where

Y1 = number of infections in location 1 (number of COVID-19 confirmed cases in county 1).

Y2 = number of infections in location 2 (number of COVID-19 confirmed cases in county 2).

X1 = determinant of Y1 (explanatory variable in county 1).

X2 = determinant of Y2 (explanatory variable in county 2).

ε1 = error term in county 1.

ε2 = error term in county 2.

As pointed out by Lesage et al. (2008), the SDM includes most models used in applied spatial econometrics literature: i) if *γ=0*, eq. (1) becomes a spatial autoregressive (SAR) model that includes a spatial lag of infected cases from related counties, but excludes these counties’ vulnerability factors; ii) if *γ* =  − *ρβ*, it becomes a spatial error model (SEM); iii) if *γ* = 0 and *ρ* = 0, it is a non-spatial least-squares model that assumes locations’ dependent variables are independent [28].

For *K* locations, Lesage and Fisher (2008) show that eq. (1) can be rewritten as

2 $$ y={\sum}_{r=1}^k{K}_r(W){X}_r+A{(W)}^{-1}\varepsilon, $$

where *K*_*r*_(*W*) = *A*(*W*)^−1^(*I*_*n*_*β*_*r*_ + *Wθ*_*r*_) and *A*(*W*)^−1^ = (*I*_*n*_ − *ρW*)^−1^, and

$$ {X}_r=\left(\begin{array}{c}{x}_{r1}\\ {}{x}_{r2}\\ {}.\\ {}.\\ {}.\\ {}{x}_{rn}\end{array}\right) $$

Lesage et al. (2011) propose a series of metrics to interpret estimates from eq. (2); direct, indirect, and total marginal effects of the determinants of y [29]. Direct marginal effects show how each determinant (Xs) affects y while indirect effects show how each determinant affects y in neighboring counties through social interactions. The total marginal effects provide a complete picture of impacts of each variable, considering the spillover effects. The expansion of the expected value of eq. (2) is given by

3 $$ \left(\begin{array}{c}\begin{array}{c}E\left({y}_1\right)\\ {}E\left({y}_2\right)\end{array}\\ {}\begin{array}{c}\vdots \\ {}E\left({y}_n\right)\end{array}\end{array}\right)=\left(\begin{array}{c}\begin{array}{cc}{K}_r{(W)}_{11}& {K}_r{(W)}_{12}\kern0.5em \dots \kern0.5em {K}_r{(W)}_{1n}\\ {}{K}_r{(W)}_{21}& {K}_r{(W)}_{21}\kern0.5em \dots \kern0.5em {K}_r{(W)}_{2n}\end{array}\\ {}\begin{array}{cc}\vdots & \begin{array}{ccc}\vdots & \ddots & \vdots \end{array}\\ {}{K}_r{(W)}_{n1}& \begin{array}{ccc}{K}_r{(W)}_{n1}& \dots & {K}_r{(W)}_{nn}\end{array}\end{array}\end{array}\right)\left(\begin{array}{c}\begin{array}{c}{x}_{1r}\\ {}{x}_{2r}\end{array}\\ {}\begin{array}{c}\vdots \\ {}{x}_{nr}\end{array}\end{array}\right) $$

for variable *i*, the expected value of (3) takes the following form:

4 $$ E\left({y}_i\right)={\sum}_{r=1}^k\left[{K}_r{(W)}_{i1}{x}_{1r}+\dots +{K}_r{(W)}_{n1}{x}_{nr}\right] $$

Spatial direct and indirect effects are derived as follows:

i) Indirect effects: the impact on the expected value of location *i* given a change in the explanatory variable *x*_*r*_ in location *j* is

5 $$ \frac{\partial E\left({y}_i\right)}{\partial {x}_{jr}}={K}_r{(W)}_{ij} $$

where *K*_*r*_(*W*)_*ij*_ represents the *i*, *j* th element of matrix *K*_*r*_(*W*).

ii) Direct effects: the impact on the expected value of location *i*, given a change in certain variable for the same location is given by

6 $$ \frac{\partial E\left({y}_i\right)}{\partial {x}_{ir}}={K}_r{(W)}_{ii} $$

It follows that for *K* locations, there will be *K* × *n*^2^ marginal effects which might be burdensome to report on.

### Data

This study uses the COVID-19 Community Vulnerability Index (CCVI) developed by the Surgo Foundation which combines the Social Vulnerability Index (SVI) with other vulnerability factors specific to COVID-19 (Epidemiological factors, public health system capacity, and variables capturing high risk environments).

Developed by the CDC, the SVI has been applied in disaster management to help identify vulnerable people and areas. It specifies the relative vulnerability of every U.S. Census tract, and is built by ranking each of the 15 census variables from highest to lowest across all census tracts in the U.S. The tracts are then grouped into four themes (Socioeconomic Status, Household Composition and Disability, Minority Status & Language, and Housing type & Transportation).

A percentile rank was then calculated for each census tract and each theme, and percentile values range from 0 to 1, with higher values indicating greater vulnerability. For each of the four themes, the percentiles are summed up for the variables comprising each theme**.** Then the summed percentiles are ordered for each theme to determine percentile ranking that are specific to each theme [30].

The CCVI builds on the SVI and identifies communities that are vulnerable by aggregating dozens of indicators across six themes (citation: Surgo ventures).

The Surgo Foundation^1^defines “more vulnerable” communities as those that “have a limited ability to mitigate, treat, and delay transmission of a pandemic disease, and to reduce its economic and social impacts” [31]. More recently, a new version of the CCVI was developed and focuses on 40 variables covering seven core themes to account for additional factors that make a community or individual susceptible to the COVID-19 pandemic. This new and composite CCVI metric ranks each geography (state, county, or census tract) relative to one another on a 0–1 scale (0 = least vulnerable, 1 = most vulnerable), and the data is available in the Surgo Foundation website [31], Appendix D.

### Dependent and independent variables

The dependent variable is the COVID-19 confirmed cumulative cases from May 1 to December 15, 2020. Fifteen sub-periods are considered: May 1–May 15, May 16–May 31, June 1–June 15, June 16–June 30, July 1–15, July 16–31, August 1–15, August 16–31, September 1–15, September 16–30, October 1–15, October 16–31, November 1–15, November 16–30, December 1–15. For each county and each sub-period, the cumulative counts of confirmed cases by county is divided by the total population multiplied by 100,000. Data on the number of people tested for COVID-19 was retrieved from the CDC COVID Data Tracker (https://covidtracking.com/about-data/cdc-comparison).

The independent variables are the CCVI for each of the 7 themes that account for the following types of vulnerability: Socioeconomic status, Minority status & Language, Transportation, Household composition & Disability, Epidemiological factors, Healthcare system, High-risk environment, and Population density. It is a percentile-based index and ranks each county relative to one another on a 0–1 scale, with 0 indicating the least vulnerable, and 1 indicating the most vulnerable.

CCVI Theme 1: Socioeconomic Status is composed of percentile rank data for the percentage below poverty, percentage unemployed, per capita income, and percentage with no high school diploma [31].

CCVI Theme 2: Minority Status & Language include percentage of minority and percentage of those who speaks English “less than well” [31].

CCVI Theme 3: Transportation, Household Composition & Disability include persons aged 17 or younger, older than 5 with a disability, single-parent households, multiunit structures, mobile homes, crowding, no vehicle, group quarters, and access to indoor plumbing [31].

CCVI Theme 4: Epidemiological factors was selected based on the CDC guidelines and identify high risk populations as elderly adults and individuals with underlying conditions (respiratory conditions, cardiovascular conditions, obesity, diabetes, immuno-compromised, and persons aged 65 or Older) [31].

CCVI Theme 5: Healthcare System factors provide a measure of the capacity, strength, accessibility, and preparedness of the healthcare system in the context of COVID-19. Health System Capacity includes hospital beds per 100,000, intensive care unit beds per 100,000, and epidemiologist per 100,000. Healthcare accessibility includes the percentage of the population with a primary care physician. Health system strength is measured by total health expenditure and quality of care and includes the Agency for Healthcare Research and Quality’s (AHRQ) prevention quality indicator (PQI) composite, Health spending per capita, and Aggregate cost of medical care. Health system preparedness includes public health emergency preparedness funding available, health labs per 100,000, and emergency services per 100,000 [31].

CCVI Theme 6: High- Risk Environments measure the percentage of population working or living in environments with high infection risk. The variables are long-term care residents per 100,000, prisons population per 100,000, and the percentage of populations employed in high-risk industry. The CCVI considers industries where workers are in enclosed spaces for extended time, in proximity of or interacting with others, and/or exposed to diseases. Rural counties for example were particularly affected by outbreaks due to their employment dependence on industries with high-risk work conditions like the meatpacking industry [32].

CCVI Theme 7: Population Density is measured as the total number of people per unit area and represent an important factor in health outcomes.

Table 1 presents the descriptive statistics of variables used for the contiguous U.S. counties.

**Table 1:** Descriptive statistics

Variables	Observations	Mean	Standard deviation	Minimum	Maximum
Index (0 to 1)					
Socioeconomic Status	3142	0.499953	0.288811	0	1
Minority Status & Language	3142	0.499835	0.288924	0	1
Transportation, Household Composition & Disability	3142	0.499962	0.288809	0	1
Epidemiological factors	3142	0.499979	0.288811	0	1
Healthcare System factors	3142	0.499963	0.288809	0	1
High Risk- Environments	3142	0.499931	0.288816	0	1
Population Density	3142	0.500000	0.288813	0	1

## Results and discussion

### Spatial dependence

The Ordinary Least Squares (OLS) model does not allow for spillovers as it implicitly assumes that outcomes for different units (counties) are independent of each other. To assess the presence of spatial interactions between counties, several statistics are used including the Moran’s Indices which are positive and significant at 1% suggesting that the COVID-19 confirmed cases exhibit spatial correlation (see Appendix A). The Lagrange Multiplier test (LM) and the Robust Lagrange Multiplier test (Robust LM) were conducted and confirmed the presence of spatial lag or error terms in the model. As expected, OLS estimates are biased and inconsistent as the spillover parameters (*ρ* and *γ*) in eq. (1) are significantly different from zero for all sub-periods.

The SDM was then used to estimate the interaction effects and exogenous interaction effects. The results from non-spatial (OLS) and spatial (SDM) specifications models for all the sub-periods are presented below (see Appendix C).

Furthermore, the average impacts (direct, indirect, and total effects) are estimated to determine the magnitude of the change in confirmed COVID-19 cases resulting from changes in the selected vulnerability factors. The direct effects measure the impact of change in vulnerability factors in one county on the confirmed COVID-19 cases within that county, while the indirect effects (spillover effects), measure the impact of same change on confirmed COVID-19 cases from the neighboring county. The total effect is the sum of both direct and indirect effects. To compare the change in spillover effects over time, the average impacts were calculated for fifteen sub-periods (May 1 – December 15, 2020). As shown in Appendix C, the increase in some of the vulnerability factors will increase the possibility of COVID-19 cases in the target county.

Overall, the results indicate significant differences between direct and indirect effects across time and across vulnerability factors. This suggests that failing to account for spatial correlation would lead to biased estimates.

#### Socioeconomic factors

**Fig. 3:**
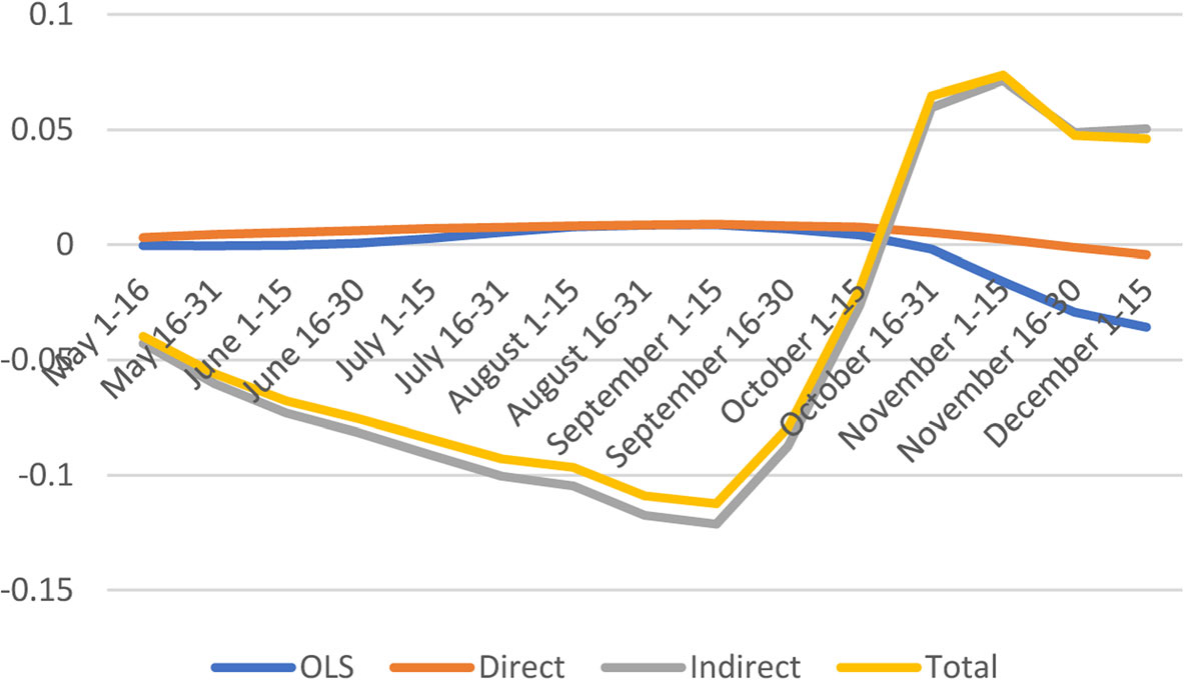
Spatial and non-spatial effects of socioeconomic status on confirmed COVID-19 cases

As expected, marginal effects of socioeconomic vulnerability on the number of confirmed cases are positive and significant; except for the last four periods indicating that COVID-19 confirmed cases increase with higher socioeconomic vulnerability (Fig. 3 and Appendix C). Similarly, Nayak found that socioeconomic status was associated with higher incidence after adjusting for age and comorbidities, and other studies found that most vulnerable counties based on socioeconomic status had greater risk of COVID-19 diagnosis and death [9, 11, 20].

Except for the last four sub-periods, the average direct effect of socioeconomic vulnerability indicates that a marginal point increase in vulnerability index is likely to increase COVID-19 cases by 0.7%. With respect to indirect effects and total effects, the socioeconomic factors appear to have negative spillover effects on the number of confirmed cases, especially early in the pandemic (May 1–June 15) and later (Sept 1 - August 31).

#### Minorities & Language

**Fig. 4:**
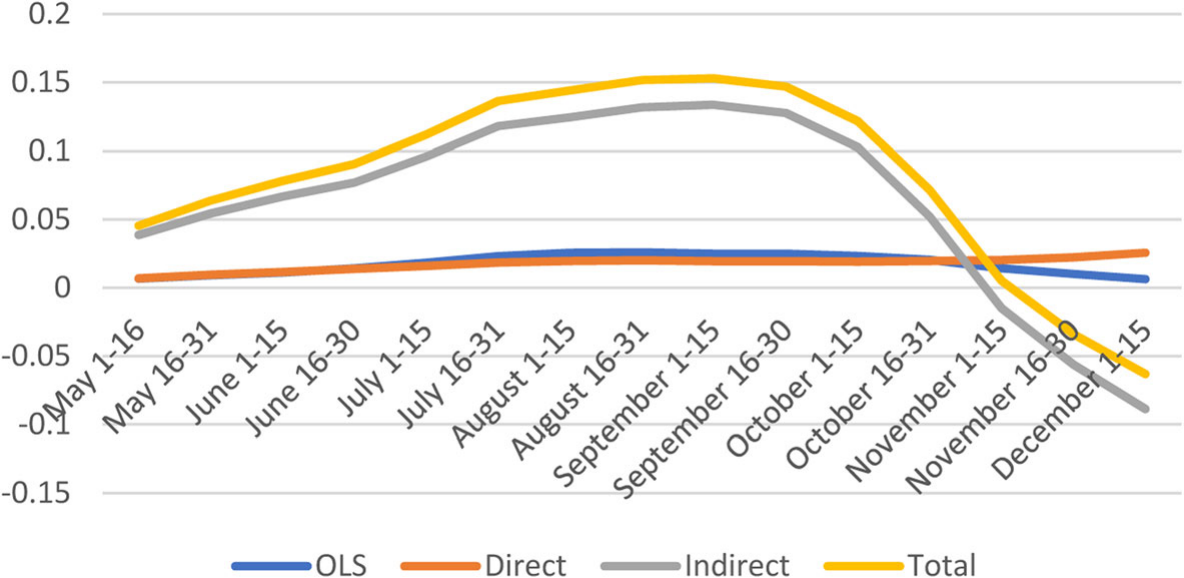
Spatial and non-spatial effects of Minority Status & Language on confirmed COVID-19 cases

The average direct effect suggest that a marginal point increase in vulnerability index is likely to increase confirmed cases in a given county by 1.7% (Fig. 4 and Appendix C).

The indirect effects of Minorities & Languages are only significant early in the pandemic and later. Additionally, the indirect effects from neighboring counties in some cases is ten times the magnitude of the direct impacts, suggesting large spillover effects. On average, the total impact is positive, with about two third comprised of the spillover effects from neighboring counties. Similar findings indicate that people in the most vulnerable counties by Minority & Language domain had greater risks of COVID-19 diagnosis [20], and increased case counts [19].

#### Housing type, Transportation, Household Composition & Disability

**Fig. 5:**
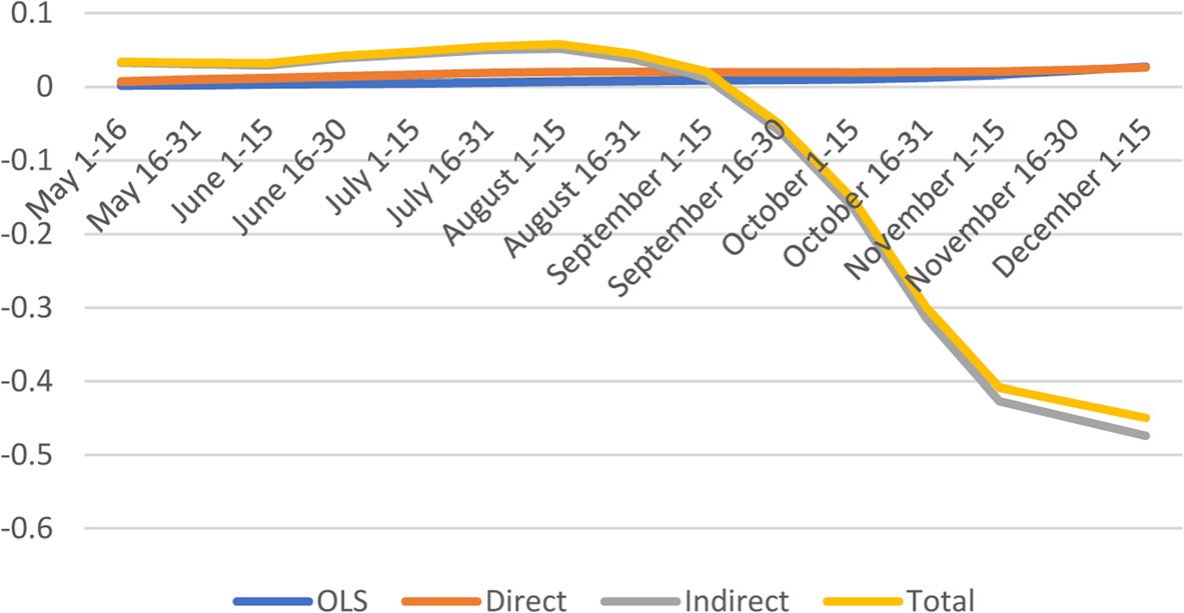
Spatial and non-spatial effects of Housing Type, Transportation, Household Composition & Disability on confirmed COVID-19 cases

The results indicate that Housing & Transportation vulnerability exert a positive direct impact on the number of confirmed cases. Counties with higher vulnerability in Housing & Transportation also experienced higher confirmed COVID-19 cases (Fig. 5). Less urban counties with high-density housing structures, and crowded housing units had a higher probability of being identified as a COVID-19 hotspot [2].

However, the indirect effects and total effects from nearby counties are negative and significant (October 1–December 15) suggesting a negative relationship between Housing & Transportation vulnerability and confirmed COVID-19 cases. Although counterintuitive, these findings are similar to those of Karaye et al. (2020) who pointed out that the direction and magnitude of social vulnerability varied among U.S. states, and perhaps could be better explained by local spatial variations between counties [19].

#### Epidemiological factors

**Fig. 6:**
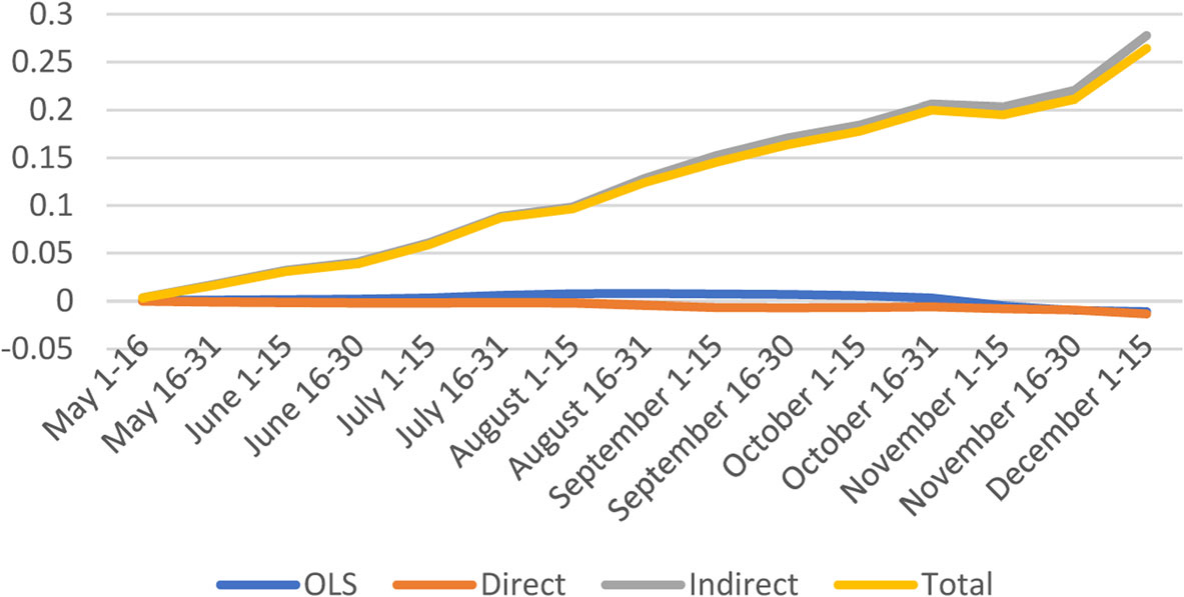
Spatial and non-spatial effects of Epidemiological Factors on confirmed COVID-19 cases

From August 16 to December 15, the direct effects of epidemiological factors are negative and significant whereas indirect effects show positive effects during the same period (Fig. 6). However, total effects are positive and significant during the same sub-periods which is partially consistent with Neelon et al. (2020) who found that the percentage in fair or poor health tested was positively associated with more COVID-19 cases; patients with comorbidities had a higher risk of mortality than patient without [11, 33].

#### Healthcare System

**Fig. 7:**
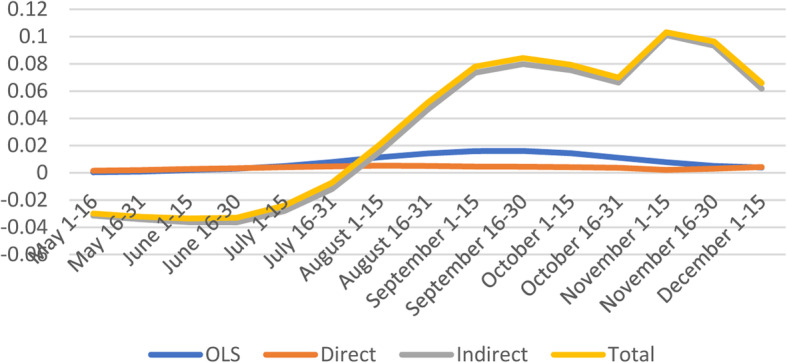
Spatial and non-spatial effects of Healthcare System Factors on confirmed COVID-19 cases

Healthcare System vulnerability factors do contribute to higher number of confirmed cases as shown by the positive and significant effects in most subperiods except during the later months of 2020 (Fig. 7). Interestingly, the indirect and total effects show significant negative coefficients early in the pandemic (May 1–June 15) and positive coefficients for the last quarter of 2020 (September 1–November 30).

The U.S health care system experienced tremendous pressure during the pandemic, and certain measures have been implemented to reduce the burden such as elective procedures, inpatient/outpatient surgery, procedures cancelled [34]. The evolution in treatment protocols has decreased dependence on ventilators, lowered overall hospital length of stay, and decreased clinical and demographic-adjusted mortality rates [35]. These measures allowed hospitals to increase inpatient bed capacity, equipment, the availability of health care providers, and decrease the risk of spread of the virus.

#### High-Risk Environments

**Fig. 8:**
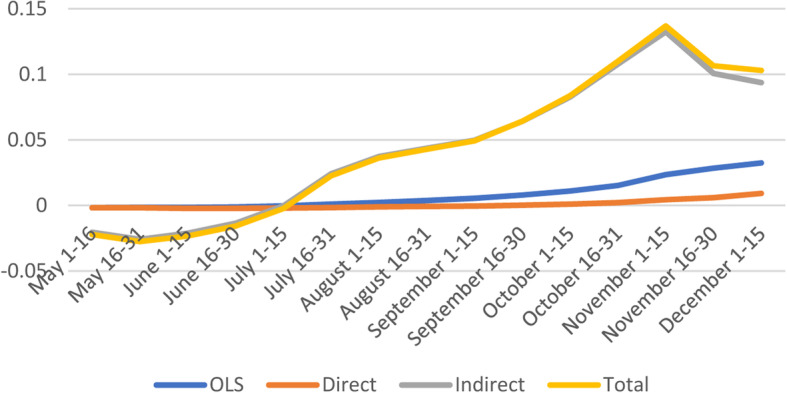
Spatial and non-spatial effects of High-Risk Environments factors on confirmed COVID-19 cases

High-Risk environment vulnerability has negative and significant direct effects coefficients early in the pandemic and turn positive later. Indirect and total effects are positive and significant from August to December (Fig. 8).

#### Population density

**Fig. 9:**
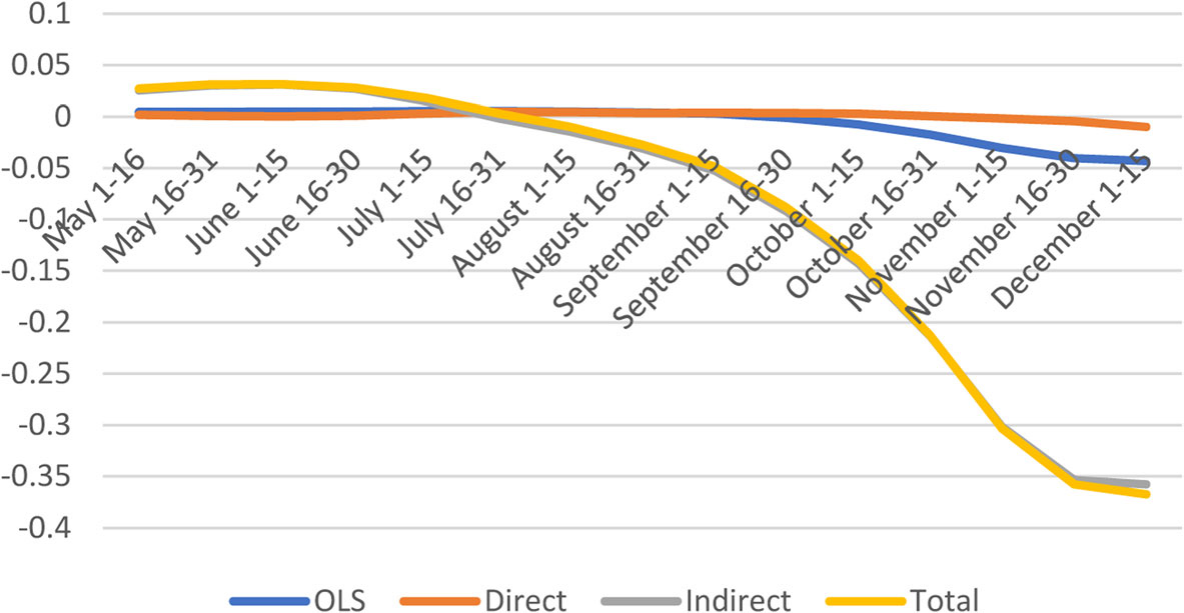
Spatial and non-spatial effects of Population Density on confirmed COVID-19 cases

The direct effects are positive and significant only during a short period (July 1–August 15), and insignificant for the remaining sub-periods. Along with other studies [36, 37], Kadi et al. (2020) used Algerian data and found a strong correlation between the population density and the number of COVID-19 infections [38]. The indirect and total effects start off positive (May 1–June 15) and turned negative during the last quarter of 2020 (Fig. 9). Studies found that population density is not the sole factor contributing to the adverse COVID-related impacts in cities [39].

## Conclusions

The purpose of this study was to assess the effects of pre-existing vulnerability factors on COVID-19 confirmed cases while accounting for spatial spillover effects. The findings show that the effect on COVID-19 cases differs widely across vulnerability factors and across counties. Consistent with previous studies, vulnerability factors such as Minorities & Language, Epidemiological factors, Transport & Housing, and Population Density are positively associated with COVID-19 confirmed cases, suggesting that higher vulnerability is associated with higher number of COVID-19 confirmed cases. The results also indicate that spatial spillover effects are important determinants of COVID-19 confirmed cases, and in some cases, these effects are higher than the direct non-spatial effects. On the other hand, direct effects estimates are on average significant and carry the expected positive sign, except for some subperiods.

A major difference with previous studies is that COVID-19 confirmed cases are examined at different points in time (March 1 – December 15) which provides a better understanding of the rapidly evolving relationship between vulnerability factors and COVID-19 confirmed cases. Although Neelon et al. (2020) used longitudinal data to examine social determinants of health and disparities in COVID-19 incidence, spatial spillovers effects were not accounted for [11]. The magnitude and direction of the relationship between vulnerability factors and COVID-19 cases vary widely among U.S. counties. Karaye et al. (2020) found that in the states of Washington and Oregon, minority status & Language as well as household composition and disability were more predictive of COVID-19 cases than housing and transportation. However, vulnerability to COVID-19 was better explained by housing and transportation than by minority status and language in other states (Gulf Coast states, Southern Arkansas, and Western Tennessee) [19].

Because of significant spillover effects, the first step in the fight against the pandemic should be a federal commitment to containing the spread of coronavirus and related fears. Using Italy’s experience, Pisano et al. (2020) notes that the most effective time to take strong action is extremely early, when the threat appears to be small — or even before there are any cases [40].

If anything, the COVID-19 crisis has taught us that pandemics precipitate much more than a global health catastrophe, countries or states cannot fix it alone, and even though pandemics have been anticipated for many years, countries were profoundly unprepared [41]. Because of potential negative spillover effects of individual actions on nearby communities, there is a need for global coalition against COVID-19 pandemic.

Moreover, the more people continue to move from one location to the other, containing infection would be challenging and would largely depend on the coordination across borders. An effective approach to controlling a pandemic such as COVID-19 should involve national/federal coordination.

One limitation of this study is that rural and urban counties were not differentiated. Indeed, counties with the highest relative pressure on healthcare system are in more rural areas of the country and have also relatively fewer resources nearby to support patients as opposed to major metropolitan areas.

## Supplementary Information

**Additional file 1.**

## Data Availability

The datasets used and/or analyzed during the current study are available from the corresponding author on reasonable request.

## Abbreviations

CDC: Center for Disease Control
SVI: Social Vulnerability Index
CCVI: COVID-19 Community Vulnerability Index
AHRQ: Agency for Healthcare Research and Quality
PQI: Prevention Quality Indicator (PQI) composite
USDA: The United States Department of Agriculture

## Footnotes

1 The Surgo Foundation collected data from the CDC, Centers for Medicare, & Medicaid Services (CMS), the Harvard Global Health Institute, PolicyMap, the U.S Bureau of Labor Statistics (BLS), the U.S. Census Bureau (USCB), the National Cancer Institute (NCI), the National Center for HIV, STD and TB Prevention (NCHSTP), Kaiser Health News, Centers for Medicare & Medicaid Services (CMS), Census Economic Annual Surveys, the Vera institute for Justice, and the Association of Public Health Laboratories.
