# Supplementary material for: Spatial spillover and COVID-19 spread in the U.S
Source: BMC Public Health. 2021 Sep 27;21:1765. doi: 10.1186/s12889-021-11809-2 (PMC8475369; doi:10.1186/s12889-021-11809-2)
Supplement: Supplementary file 1 — Additional file 1. [file 12889_2021_11809_MOESM1_ESM.docx]

**LEGEND**

**APPENDIX A. Moran Test results page 2**

**APPENDIX B. Estimation Results – Spatial Durbin Model (SDM) page 3**

**APPENDIX C. Estimation Results – OLS, Direct, Indirect and Total Effects page 18**

**APPENDIX D. COVID-19 Community Vulnerability Index (CCVI) page 25**

**APPENDIX**

**APPENDIX A. Moran Test results**

| **Time** | **Global Moran MI** | | **Morean MI Error Test** | |
| --- | --- | --- | --- | --- |
|  | **Coefficient** | **z stat** | **Coefficient** | **z stat** |
| May 1-16 | 0.0314 *** | 50.3 | 81.5 *** | 130000 |
| May 16-31 | 0.0341 *** | 54.2 | 88.4 *** | 140000 |
| June 1-15 | 0.034 *** | 53.9 | 88.0 *** | 140000 |
| June 16-30 | 0.035 *** | 55.4 | 90.7 *** | 140000 |
| July 1-15 | 0.0388 *** | 61.2 | 100.4 *** | 160000 |
| July 16-31 | 0.0507 *** | 79.6 | 130.8 *** | 200000 |
| August 1-15 | 0.0598 *** | 93.7 | 154.2 *** | 240000 |
| August 16-31 | 0.0663 *** | 103.9 | 170.9 *** | 270000 |
| September 1-15 | 0.0703 *** | 110.1 | 181.1 *** | 280000 |
| September 16-30 | 0.0739 *** | 115.6 | 190.3 *** | 300000 |
| October 1-15 | 0.078 *** | 121.9 | 200.7 *** | 310000 |
| October 16-31 | 0.0797 *** | 124.5 | 205.1 *** | 320000 |
| November 1-15 | 0.0883 *** | 137.8 | 227.1 *** | 350000 |
| November 16-30 | 0.0911 *** | 142.1 | 234.2 *** | 360000 |
| December 1-15 | 0.0885 *** | 138.1 | 227.6 *** | 350000 |

*, **, *** denote statistical significance at the 10%, 5%, and 1% levels, respectively.

**APPENDIX B. Estimation Results – Spatial Durbin Model (SDM)**

| Confirmed cases (May 1-16) | Coef. | Std. Err. | z | P>z | [95% Conf. | | Interval] | | |
| --- | --- | --- | --- | --- | --- | --- | --- | --- | --- |
| Socioeconomics | 0.003 | 0.001 | 3.000 | 0.003 | 0.001 | | 0.005 | | |
| Minorities & Language | 0.007 | 0.001 | 7.570 | 0.000 | 0.005 | | 0.009 | | |
| Transp, Housing, Household & Disabil. | 0.001 | 0.001 | 1.450 | 0.146 | 0.000 | | 0.003 | | |
| Epidemiology | 0.000 | 0.001 | -0.210 | 0.836 | -0.002 | | 0.002 | | |
| Health Care System | 0.001 | 0.001 | 1.810 | 0.071 | 0.000 | | 0.003 | | |
| High-Risk Environment | -0.002 | 0.001 | -2.630 | 0.009 | -0.003 | | 0.000 | | |
| Pop Density | 0.002 | 0.001 | 1.810 | 0.070 | 0.000 | | 0.004 | | |
| _cons | -0.697 | 0.001 | -594.890 | 0.000 | -0.699 | | -0.695 | | |
| W |  |  |  |  |  | |  | | |
| W*Socioeconomics | -0.065 | 0.023 | -2.830 | 0.005 | -0.111 | | -0.020 | | |
| W*Minorities & Language | 0.059 | 0.013 | 4.450 | 0.000 | 0.033 | | 0.085 | | |
| W*Transp, Housing, Household & Disabil. | 0.049 | 0.022 | 2.280 | 0.023 | 0.007 | | 0.091 | | |
| W*Epidemiology | 0.005 | 0.021 | 0.250 | 0.806 | -0.035 | | 0.045 | | |
| W*Health Care System | -0.048 | 0.015 | -3.220 | 0.001 | -0.077 | | -0.019 | | |
| W*High-Risk Environment | -0.031 | 0.017 | -1.870 | 0.061 | -0.064 | | 0.001 | | |
| W*Pop Density | 0.039 | 0.007 | 5.420 | 0.000 | 0.025 | | 0.053 | | |
| Rho | -0.023 | 0.003 | -8.140 | 0.000 | -0.028 | | -0.017 | | |
| Confirmed cases (May 16-31) | Coef. | Std. Err. | z | P>z | | [95% Conf. | | Interval] |  |
| Socioeconomics | 0.004 | 0.001 | 3.710 | 0.000 | | 0.002 | | 0.007 |  |
| Minorities & Language | 0.009 | 0.001 | 9.030 | 0.000 | | 0.007 | | 0.011 |  |
| Transp, Housing, Household & Disabil. | 0.002 | 0.001 | 1.960 | 0.050 | | 0.000 | | 0.004 |  |
| Epidemiology | -0.001 | 0.001 | -1.030 | 0.305 | | -0.003 | | 0.001 |  |
| Health Care System | 0.002 | 0.001 | 2.040 | 0.041 | | 0.000 | | 0.004 |  |
| High-Risk Environment | -0.002 | 0.001 | -2.280 | 0.022 | | -0.003 | | 0.000 |  |
| Pop Density | 0.001 | 0.001 | 0.730 | 0.466 | | -0.001 | | 0.003 |  |
| Cons | -0.698 | 0.001 | -517.150 | 0.000 | | -0.701 | | -0.696 |  |
| W |  |  |  |  | |  | |  |  |
| W*Socioeconomics | -0.093 | 0.027 | -3.480 | 0.001 | | -0.145 | | -0.040 |  |
| W*Minorities & Language | 0.084 | 0.015 | 5.470 | 0.000 | | 0.054 | | 0.114 |  |
| W*Transp, Housing, Household & Disabil. | 0.046 | 0.025 | 1.870 | 0.062 | | -0.002 | | 0.095 |  |
| W*Epidemiology | 0.027 | 0.024 | 1.120 | 0.263 | | -0.020 | | 0.073 |  |
| W*Health Care System | -0.052 | 0.017 | -3.050 | 0.002 | | -0.086 | | -0.019 |  |
| W*High-Risk Environment | -0.040 | 0.019 | -2.060 | 0.039 | | -0.077 | | -0.002 |  |
| W*Pop Density | 0.046 | 0.008 | 5.650 | 0.000 | | 0.030 | | 0.062 |  |
| Rho | -0.031 | 0.003 | -9.430 | 0.000 | | -0.037 | | -0.024 |  |

| Confirmed cases (June 1-15) | Coef. | Std. Err. | z | P>z | [95% Conf. | Interval] |
| --- | --- | --- | --- | --- | --- | --- |
| Socioeconomics | 0.005 | 0.001 | 3.890 | 0.000 | 0.003 | 0.008 |
| Minorities & Language | 0.011 | 0.001 | 9.560 | 0.000 | 0.009 | 0.014 |
| Transp, Housing, Household & Disabil. | 0.003 | 0.001 | 2.510 | 0.012 | 0.001 | 0.005 |
| Epidemiology | -0.002 | 0.001 | -1.310 | 0.190 | -0.004 | 0.001 |
| Health Care System | 0.003 | 0.001 | 2.540 | 0.011 | 0.001 | 0.005 |
| High-Risk Environment | -0.002 | 0.001 | -2.470 | 0.013 | -0.004 | 0.000 |
| Pop Density | 0.000 | 0.001 | 0.300 | 0.765 | -0.002 | 0.003 |
| Cons | -0.698 | 0.002 | -448.580 | 0.000 | -0.701 | -0.695 |
| W |  |  |  |  |  |  |
| W*Socioeconomics | -0.112 | 0.031 | -3.660 | 0.000 | -0.173 | -0.052 |
| W*Minorities & Language | 0.103 | 0.018 | 5.840 | 0.000 | 0.069 | 0.138 |
| W*Transp, Housing, Household & Disabil. | 0.044 | 0.029 | 1.540 | 0.124 | -0.012 | 0.100 |
| W*Epidemiology | 0.050 | 0.027 | 1.820 | 0.069 | -0.004 | 0.103 |
| W*Health Care System | -0.056 | 0.020 | -2.820 | 0.005 | -0.095 | -0.017 |
| W*High-Risk Environment | -0.033 | 0.022 | -1.470 | 0.141 | -0.076 | 0.011 |
| W*Pop Density | 0.048 | 0.009 | 5.030 | 0.000 | 0.029 | 0.066 |
| Rho | -0.035 | 0.004 | -9.330 | 0.000 | -0.043 | -0.028 |

| Confirmed cases (June 16-30) | Coef. | Std. Err. | z | P>z | [95% Conf. | Interval] |
| --- | --- | --- | --- | --- | --- | --- |
| Socioeconomics | 0.006 | 0.001 | 4.170 | 0.000 | 0.003 | 0.009 |
| Minorities & Language | 0.014 | 0.001 | 10.610 | 0.000 | 0.011 | 0.016 |
| Transp, Housing, Household & Disabil. | 0.003 | 0.001 | 2.690 | 0.007 | 0.001 | 0.006 |
| Epidemiology | -0.002 | 0.001 | -1.510 | 0.130 | -0.005 | 0.001 |
| Health Care System | 0.003 | 0.001 | 2.840 | 0.005 | 0.001 | 0.006 |
| High-Risk Environment | -0.002 | 0.001 | -2.290 | 0.022 | -0.004 | 0.000 |
| Pop Density | 0.001 | 0.001 | 0.700 | 0.482 | -0.002 | 0.004 |
| Cons | -0.696 | 0.002 | -414.790 | 0.000 | -0.699 | -0.693 |
| W |  |  |  |  |  |  |
| W*Socioeconomics | -0.126 | 0.033 | -3.800 | 0.000 | -0.190 | -0.061 |
| W*Minorities & Language | 0.119 | 0.019 | 6.270 | 0.000 | 0.082 | 0.157 |
| W*Transp, Housing, Household & Disabil. | 0.059 | 0.031 | 1.910 | 0.056 | -0.001 | 0.119 |
| W*Epidemiology | 0.063 | 0.029 | 2.140 | 0.033 | 0.005 | 0.121 |
| W*Health Care System | -0.056 | 0.021 | -2.630 | 0.009 | -0.098 | -0.014 |
| W*High-Risk Environment | -0.021 | 0.024 | -0.880 | 0.380 | -0.068 | 0.026 |
| W*Pop Density | 0.042 | 0.010 | 4.090 | 0.000 | 0.022 | 0.062 |
| Rho | -0.038 | 0.004 | -9.310 | 0.000 | -0.046 | -0.030 |

| Confirmed cases (July 1-15) | Coef. | Std. Err. | z | P>z | [95% Conf. | Interval] |
| --- | --- | --- | --- | --- | --- | --- |
| Socioeconomics | 0.007 | 0.002 | 4.390 | 0.000 | 0.004 | 0.010 |
| Minorities & Language | 0.016 | 0.001 | 11.390 | 0.000 | 0.013 | 0.019 |
| Transp, Housing, Household & Disabil. | 0.004 | 0.001 | 2.860 | 0.004 | 0.001 | 0.006 |
| Epidemiology | -0.002 | 0.001 | -1.420 | 0.157 | -0.005 | 0.001 |
| Health Care System | 0.004 | 0.001 | 3.180 | 0.001 | 0.002 | 0.007 |
| High-Risk Environment | -0.002 | 0.001 | -1.980 | 0.048 | -0.004 | 0.000 |
| Pop Density | 0.003 | 0.002 | 2.130 | 0.033 | 0.000 | 0.006 |
| Cons | -0.691 | 0.002 | -379.800 | 0.000 | -0.694 | -0.687 |
| W |  |  |  |  |  |  |
| W*Socioeconomics | -0.140 | 0.036 | -3.920 | 0.000 | -0.211 | -0.070 |
| W*Minorities & Language | 0.149 | 0.021 | 7.210 | 0.000 | 0.108 | 0.189 |
| W*Transp, Housing, Household & Disabil. | 0.068 | 0.033 | 2.020 | 0.043 | 0.002 | 0.133 |
| W*Epidemiology | 0.094 | 0.032 | 2.950 | 0.003 | 0.032 | 0.157 |
| W*Health Care System | -0.043 | 0.023 | -1.870 | 0.061 | -0.089 | 0.002 |
| W*High-Risk Environment | -0.001 | 0.026 | -0.020 | 0.984 | -0.051 | 0.050 |
| W*Pop Density | 0.023 | 0.011 | 2.070 | 0.039 | 0.001 | 0.044 |
| Rho | -0.039 | 0.004 | -8.830 | 0.000 | -0.048 | -0.031 |

| Confirmed cases (July 16-31) | Coef. | Std. Err. | z | P>z | [95% Conf. | Interval] |
| --- | --- | --- | --- | --- | --- | --- |
| Socioeconomics | 0.008 | 0.002 | 4.300 | 0.000 | 0.004 | 0.011 |
| Minorities & Language | 0.018 | 0.002 | 11.800 | 0.000 | 0.015 | 0.021 |
| Transp, Housing, Household & Disabil. | 0.005 | 0.001 | 3.350 | 0.001 | 0.002 | 0.008 |
| Epidemiology | -0.002 | 0.002 | -1.180 | 0.237 | -0.005 | 0.001 |
| Health Care System | 0.005 | 0.001 | 3.240 | 0.001 | 0.002 | 0.007 |
| High-Risk Environment | -0.002 | 0.001 | -1.430 | 0.153 | -0.004 | 0.001 |
| Pop Density | 0.005 | 0.002 | 2.780 | 0.005 | 0.001 | 0.008 |
| Cons | -0.683 | 0.002 | -337.990 | 0.000 | -0.687 | -0.679 |
| W |  |  |  |  |  |  |
| W*Socioeconomics | -0.155 | 0.040 | -3.890 | 0.000 | -0.233 | -0.077 |
| W*Minorities & Language | 0.183 | 0.023 | 7.990 | 0.000 | 0.138 | 0.228 |
| W*Transp, Housing, Household & Disabil. | 0.077 | 0.037 | 2.060 | 0.039 | 0.004 | 0.149 |
| W*Epidemiology | 0.137 | 0.035 | 3.870 | 0.000 | 0.068 | 0.207 |
| W*Health Care System | -0.018 | 0.026 | -0.710 | 0.480 | -0.069 | 0.032 |
| W*High-Risk Environment | 0.037 | 0.029 | 1.300 | 0.193 | -0.019 | 0.094 |
| W*Pop Density | -0.002 | 0.012 | -0.170 | 0.863 | -0.026 | 0.022 |
| Rho | -0.041 | 0.005 | -8.270 | 0.000 | -0.051 | -0.031 |

| Confirmed cases (Aug. 1-15) | Coef. | Std. Err. | z | P>z | [95% Conf. | Interval] |
| --- | --- | --- | --- | --- | --- | --- |
| Socioeconomics | 0.008 | 0.002 | 4.150 | 0.000 | 0.004 | 0.012 |
| Minorities & Language | 0.020 | 0.002 | 11.290 | 0.000 | 0.016 | 0.023 |
| Transp, Housing, Household & Disabil. | 0.006 | 0.002 | 3.720 | 0.000 | 0.003 | 0.009 |
| Epidemiology | -0.002 | 0.002 | -1.180 | 0.236 | -0.006 | 0.001 |
| Health Care System | 0.005 | 0.002 | 3.290 | 0.001 | 0.002 | 0.008 |
| High-Risk Environment | -0.001 | 0.001 | -0.880 | 0.379 | -0.004 | 0.001 |
| Pop Density | 0.005 | 0.002 | 2.430 | 0.015 | 0.001 | 0.008 |
| Cons | -0.678 | 0.002 | -300.830 | 0.000 | -0.682 | -0.673 |
| W |  |  |  |  |  |  |
| W*Socioeconomics | -0.162 | 0.044 | -3.640 | 0.000 | -0.249 | -0.075 |
| W*Minorities & Language | 0.194 | 0.026 | 7.570 | 0.000 | 0.144 | 0.244 |
| W*Transp, Housing, Household & Disabil. | 0.080 | 0.041 | 1.920 | 0.054 | -0.001 | 0.161 |
| W*Epidemiology | 0.153 | 0.040 | 3.860 | 0.000 | 0.075 | 0.230 |
| W*Health Care System | 0.024 | 0.029 | 0.850 | 0.398 | -0.032 | 0.080 |
| W*High-Risk Environment | 0.058 | 0.032 | 1.800 | 0.071 | -0.005 | 0.121 |
| W*Pop Density | -0.022 | 0.014 | -1.620 | 0.105 | -0.049 | 0.005 |
| Rho | -0.044 | 0.006 | -7.760 | 0.000 | -0.055 | -0.033 |

| Confirmed cases (Aug. 16-31) | Coef. | Std. Err. | z | P>z | [95% Conf. | Interval] |
| --- | --- | --- | --- | --- | --- | --- |
| Socioeconomics | 0.008 | 0.002 | 3.970 | 0.000 | 0.004 | 0.013 |
| Minorities & Language | 0.020 | 0.002 | 10.540 | 0.000 | 0.016 | 0.024 |
| Transp, Housing, Household & Disabil. | 0.007 | 0.002 | 4.000 | 0.000 | 0.004 | 0.011 |
| Epidemiology | -0.004 | 0.002 | -2.300 | 0.022 | -0.008 | -0.001 |
| Health Care System | 0.005 | 0.002 | 2.910 | 0.004 | 0.002 | 0.008 |
| High-Risk Environment | -0.001 | 0.001 | -0.540 | 0.590 | -0.004 | 0.002 |
| Pop Density | 0.004 | 0.002 | 1.830 | 0.067 | 0.000 | 0.008 |
| Cons | -0.675 | 0.002 | -274.160 | 0.000 | -0.680 | -0.671 |
| W |  |  |  |  |  |  |
| W*Socioeconomics | -0.182 | 0.049 | -3.750 | 0.000 | -0.278 | -0.087 |
| W*Minorities & Language | 0.206 | 0.028 | 7.360 | 0.000 | 0.151 | 0.261 |
| W*Transp, Housing, Household & Disabil. | 0.058 | 0.045 | 1.280 | 0.201 | -0.031 | 0.147 |
| W*Epidemiology | 0.200 | 0.043 | 4.620 | 0.000 | 0.115 | 0.284 |
| W*Health Care System | 0.072 | 0.031 | 2.310 | 0.021 | 0.011 | 0.134 |
| W*High-Risk Environment | 0.068 | 0.035 | 1.940 | 0.053 | -0.001 | 0.137 |
| W*Pop Density | -0.048 | 0.015 | -3.200 | 0.001 | -0.077 | -0.019 |
| Rho | -0.049 | 0.006 | -8.010 | 0.000 | -0.062 | -0.037 |

| Confirmed cases (Sept. 1-15) | Coef. | Std. Err. | z | P>z | [95% Conf. | Interval] |
| --- | --- | --- | --- | --- | --- | --- |
| Socioeconomics | 0.009 | 0.002 | 3.880 | 0.000 | 0.004 | 0.013 |
| Minorities & Language | 0.019 | 0.002 | 9.540 | 0.000 | 0.015 | 0.023 |
| Transp, Housing, Household & Disabil. | 0.008 | 0.002 | 4.220 | 0.000 | 0.004 | 0.012 |
| Epidemiology | -0.007 | 0.002 | -3.290 | 0.001 | -0.011 | -0.003 |
| Health Care System | 0.004 | 0.002 | 2.430 | 0.015 | 0.001 | 0.008 |
| High-Risk Environment | 0.000 | 0.002 | -0.320 | 0.750 | -0.003 | 0.002 |
| Pop Density | 0.004 | 0.002 | 1.720 | 0.085 | -0.001 | 0.008 |
| Cons | -0.674 | 0.003 | -255.480 | 0.000 | -0.679 | -0.669 |
| W |  |  |  |  |  |  |
| W*Socioeconomics | -0.189 | 0.052 | -3.630 | 0.000 | -0.291 | -0.087 |
| W*Minorities & Language | 0.209 | 0.030 | 7.000 | 0.000 | 0.151 | 0.268 |
| W*Transp, Housing, Household & Disabil. | 0.019 | 0.048 | 0.400 | 0.692 | -0.076 | 0.114 |
| W*Epidemiology | 0.237 | 0.046 | 5.130 | 0.000 | 0.147 | 0.328 |
| W*Health Care System | 0.115 | 0.033 | 3.430 | 0.001 | 0.049 | 0.180 |
| W*High-Risk Environment | 0.078 | 0.038 | 2.070 | 0.038 | 0.004 | 0.151 |
| W*Pop Density | -0.081 | 0.016 | -5.080 | 0.000 | -0.113 | -0.050 |
| Rho | -0.055 | 0.007 | -8.220 | 0.000 | -0.068 | -0.042 |

| Confirmed cases (Sept. 16-30) | Coef. | Std. Err. | z | P>z | [95% Conf. | Interval] |
| --- | --- | --- | --- | --- | --- | --- |
| Socioeconomics | 0.008 | 0.002 | 3.250 | 0.001 | 0.003 | 0.013 |
| Minorities & Language | 0.019 | 0.002 | 8.800 | 0.000 | 0.015 | 0.024 |
| Transp, Housing, Household & Disabil. | 0.010 | 0.002 | 4.630 | 0.000 | 0.006 | 0.014 |
| Epidemiology | -0.007 | 0.002 | -3.140 | 0.002 | -0.011 | -0.003 |
| Health Care System | 0.004 | 0.002 | 2.220 | 0.027 | 0.001 | 0.008 |
| High-Risk Environment | 0.000 | 0.002 | 0.090 | 0.925 | -0.003 | 0.003 |
| Pop Density | 0.004 | 0.002 | 1.520 | 0.128 | -0.001 | 0.008 |
| Cons | -0.672 | 0.003 | -234.400 | 0.000 | -0.677 | -0.666 |
| W |  |  |  |  |  |  |
| W*Socioeconomics | -0.136 | 0.056 | -2.410 | 0.016 | -0.247 | -0.026 |
| W*Minorities & Language | 0.201 | 0.032 | 6.180 | 0.000 | 0.137 | 0.264 |
| W*Transp, Housing, Household & Disabil. | -0.094 | 0.053 | -1.790 | 0.073 | -0.197 | 0.009 |
| W*Epidemiology | 0.267 | 0.050 | 5.320 | 0.000 | 0.169 | 0.365 |
| W*Health Care System | 0.125 | 0.036 | 3.440 | 0.001 | 0.054 | 0.196 |
| W*High-Risk Environment | 0.100 | 0.041 | 2.460 | 0.014 | 0.021 | 0.180 |
| W*Pop Density | -0.144 | 0.017 | -8.300 | 0.000 | -0.178 | -0.110 |
| Rho | -0.058 | 0.007 | -8.010 | 0.000 | -0.073 | -0.044 |

| Confirmed cases (Oct. 1-15) | Coef. | Std. Err. | z | P>z | [95% Conf. | Interval] |
| --- | --- | --- | --- | --- | --- | --- |
| Socioeconomics | 0.008 | 0.003 | 2.760 | 0.006 | 0.002 | 0.013 |
| Minorities & Language | 0.019 | 0.002 | 7.820 | 0.000 | 0.014 | 0.024 |
| Transp, Housing, Household & Disabil. | 0.012 | 0.002 | 5.110 | 0.000 | 0.007 | 0.016 |
| Epidemiology | -0.007 | 0.002 | -2.700 | 0.007 | -0.011 | -0.002 |
| Health Care System | 0.004 | 0.002 | 1.820 | 0.068 | 0.000 | 0.008 |
| High-Risk Environment | 0.001 | 0.002 | 0.560 | 0.575 | -0.003 | 0.005 |
| Pop Density | 0.003 | 0.003 | 1.110 | 0.269 | -0.002 | 0.008 |
| Cons | -0.668 | 0.003 | -211.740 | 0.000 | -0.675 | -0.662 |
| W |  |  |  |  |  |  |
| W*Socioeconomics | -0.041 | 0.062 | -0.660 | 0.512 | -0.163 | 0.081 |
| W*Minorities & Language | 0.162 | 0.036 | 4.520 | 0.000 | 0.092 | 0.232 |
| W*Transp, Housing, Household & Disabil. | -0.257 | 0.058 | -4.440 | 0.000 | -0.370 | -0.143 |
| W*Epidemiology | 0.288 | 0.055 | 5.220 | 0.000 | 0.180 | 0.397 |
| W*Health Care System | 0.118 | 0.040 | 2.950 | 0.003 | 0.040 | 0.196 |
| W*High-Risk Environment | 0.129 | 0.045 | 2.880 | 0.004 | 0.041 | 0.217 |
| W*Pop Density | -0.224 | 0.019 | -11.730 | 0.000 | -0.261 | -0.186 |
| Rho | -0.057 | 0.008 | -7.090 | 0.000 | -0.073 | -0.042 |

| Confirmed cases (Oct. 16-31) | Coef. | Std. Err. | z | P>z | [95% Conf. | Interval] |
| --- | --- | --- | --- | --- | --- | --- |
| Socioeconomics | 0.005 | 0.003 | 1.640 | 0.101 | -0.001 | 0.011 |
| Minorities & Language | 0.020 | 0.003 | 7.020 | 0.000 | 0.014 | 0.025 |
| Transp, Housing, Household & Disabil. | 0.015 | 0.003 | 5.690 | 0.000 | 0.010 | 0.020 |
| Epidemiology | -0.006 | 0.003 | -2.130 | 0.033 | -0.012 | 0.000 |
| Health Care System | 0.004 | 0.003 | 1.440 | 0.151 | -0.001 | 0.009 |
| High-Risk Environment | 0.002 | 0.002 | 1.040 | 0.300 | -0.002 | 0.006 |
| Pop Density | 0.000 | 0.003 | 0.140 | 0.889 | -0.005 | 0.006 |
| Cons | -0.662 | 0.004 | -182.330 | 0.000 | -0.669 | -0.655 |
| W |  |  |  |  |  |  |
| W*Socioeconomics | 0.093 | 0.071 | 1.300 | 0.193 | -0.047 | 0.233 |
| W*Minorities & Language | 0.082 | 0.041 | 2.000 | 0.046 | 0.001 | 0.162 |
| W*Transp, Housing, Household & Disabil. | -0.488 | 0.066 | -7.350 | 0.000 | -0.619 | -0.358 |
| W*Epidemiology | 0.320 | 0.063 | 5.040 | 0.000 | 0.196 | 0.444 |
| W*Health Care System | 0.103 | 0.046 | 2.250 | 0.025 | 0.013 | 0.193 |
| W*High-Risk Environment | 0.168 | 0.052 | 3.250 | 0.001 | 0.067 | 0.269 |
| W*Pop Density | -0.332 | 0.022 | -15.170 | 0.000 | -0.375 | -0.289 |
| Rho | -0.050 | 0.009 | -5.250 | 0.000 | -0.068 | -0.031 |

| Confirmed cases (Nov. 1-15) | Coef. | Std. Err. | z | P>z | [95% Conf. | Interval] |
| --- | --- | --- | --- | --- | --- | --- |
| Socioeconomics | 0.002 | 0.004 | 0.630 | 0.529 | -0.005 | 0.009 |
| Minorities & Language | 0.020 | 0.003 | 6.320 | 0.000 | 0.014 | 0.026 |
| Transp, Housing, Household & Disabil. | 0.018 | 0.003 | 6.120 | 0.000 | 0.012 | 0.024 |
| Epidemiology | -0.008 | 0.003 | -2.490 | 0.013 | -0.014 | -0.002 |
| Health Care System | 0.002 | 0.003 | 0.720 | 0.470 | -0.004 | 0.008 |
| High-Risk Environment | 0.004 | 0.002 | 1.860 | 0.062 | 0.000 | 0.009 |
| Pop Density | -0.002 | 0.003 | -0.510 | 0.611 | -0.008 | 0.005 |
| Cons | -0.656 | 0.004 | -157.770 | 0.000 | -0.664 | -0.648 |
| W |  |  |  |  |  |  |
| W*Socioeconomics | 0.111 | 0.082 | 1.360 | 0.174 | -0.049 | 0.271 |
| W*Minorities & Language | -0.022 | 0.047 | -0.480 | 0.633 | -0.114 | 0.070 |
| W*Transp, Housing, Household & Disabil. | -0.664 | 0.076 | -8.730 | 0.000 | -0.813 | -0.515 |
| W*Epidemiology | 0.316 | 0.073 | 4.340 | 0.000 | 0.173 | 0.458 |
| W*Health Care System | 0.157 | 0.053 | 3.000 | 0.003 | 0.054 | 0.260 |
| W*High-Risk Environment | 0.207 | 0.059 | 3.500 | 0.000 | 0.091 | 0.322 |
| W*Pop Density | -0.470 | 0.025 | -18.850 | 0.000 | -0.519 | -0.421 |
| Rho | -0.051 | 0.011 | -4.640 | 0.000 | -0.073 | -0.030 |

| Confirmed cases (Nov. 16-30) | Coef. | Std. Err. | z | P>z | [95% Conf. | Interval] |
| --- | --- | --- | --- | --- | --- | --- |
| Socioeconomics | -0.001 | 0.004 | -0.300 | 0.762 | -0.009 | 0.006 |
| Minorities & Language | 0.022 | 0.003 | 6.400 | 0.000 | 0.015 | 0.029 |
| Transp, Housing, Household & Disabil. | 0.021 | 0.003 | 6.540 | 0.000 | 0.015 | 0.028 |
| Epidemiology | -0.009 | 0.004 | -2.620 | 0.009 | -0.016 | -0.002 |
| Health Care System | 0.003 | 0.003 | 0.940 | 0.345 | -0.003 | 0.009 |
| High-Risk Environment | 0.006 | 0.003 | 2.300 | 0.022 | 0.001 | 0.011 |
| Pop Density | -0.004 | 0.004 | -1.160 | 0.246 | -0.012 | 0.003 |
| Cons | -0.651 | 0.005 | -143.450 | 0.000 | -0.660 | -0.642 |
| W |  |  |  |  |  |  |
| W*Socioeconomics | 0.077 | 0.089 | 0.860 | 0.391 | -0.098 | 0.251 |
| W*Minorities & Language | -0.087 | 0.051 | -1.700 | 0.089 | -0.187 | 0.013 |
| W*Transp, Housing, Household & Disabil. | -0.707 | 0.083 | -8.530 | 0.000 | -0.870 | -0.545 |
| W*Epidemiology | 0.346 | 0.079 | 4.370 | 0.000 | 0.191 | 0.501 |
| W*Health Care System | 0.147 | 0.057 | 2.570 | 0.010 | 0.035 | 0.259 |
| W*High-Risk Environment | 0.159 | 0.064 | 2.470 | 0.014 | 0.033 | 0.285 |
| W*Pop Density | -0.557 | 0.027 | -20.510 | 0.000 | -0.610 | -0.503 |
| Rho | -0.066 | 0.012 | -5.350 | 0.000 | -0.090 | -0.042 |

| Confirmed cases (Dec. 1-15) | Coef. | Std. Err. | z | P>z | [95% Conf. | Interval] |
| --- | --- | --- | --- | --- | --- | --- |
| Socioeconomics | -0.004 | 0.004 | -1.040 | 0.300 | -0.013 | 0.004 |
| Minorities & Language | 0.026 | 0.004 | 6.890 | 0.000 | 0.018 | 0.033 |
| Transp, Housing, Household & Disabil. | 0.025 | 0.004 | 7.010 | 0.000 | 0.018 | 0.031 |
| Epidemiology | -0.013 | 0.004 | -3.560 | 0.000 | -0.021 | -0.006 |
| Health Care System | 0.004 | 0.003 | 1.220 | 0.221 | -0.002 | 0.011 |
| High-Risk Environment | 0.009 | 0.003 | 3.340 | 0.001 | 0.004 | 0.015 |
| Pop Density | -0.010 | 0.004 | -2.440 | 0.015 | -0.018 | -0.002 |
| Cons | -0.637 | 0.005 | -130.820 | 0.000 | -0.646 | -0.627 |
| W |  |  |  |  |  |  |
| W*Socioeconomics | 0.079 | 0.096 | 0.830 | 0.407 | -0.108 | 0.267 |
| W*Minorities & Language | -0.138 | 0.055 | -2.530 | 0.012 | -0.246 | -0.031 |
| W*Transp, Housing, Household & Disabil. | -0.745 | 0.089 | -8.380 | 0.000 | -0.920 | -0.571 |
| W*Epidemiology | 0.437 | 0.085 | 5.140 | 0.000 | 0.270 | 0.603 |
| W*Health Care System | 0.098 | 0.061 | 1.590 | 0.112 | -0.023 | 0.218 |
| W*High-Risk Environment | 0.148 | 0.069 | 2.150 | 0.032 | 0.013 | 0.284 |
| W*Pop Density | -0.565 | 0.029 | -19.410 | 0.000 | -0.622 | -0.508 |
| Rho | -0.069 | 0.014 | -5.040 | 0.000 | -0.096 | -0.042 |

**APPENDIX C. Estimation Results – OLS, Direct, Indirect and Total Effects**

| SOCIO | non-spatial | | Spatial Durbin Model | | | | | |
| --- | --- | --- | --- | --- | --- | --- | --- | --- |
|  | **Direct - OLS** | | **Direct** | | **Indirect** | | **Total** | |
|  | **Coeff.** | **Sig.** | **Coeff.** | **Sig.** | **Coeff.** | **Sig.** | **Coeff.** | **Sig.** |
| May 1-16 | -0.001 |  | 0.003 | ** | -0.043 | ** | -0.040 | * |
| May 16-31 | -0.001 |  | 0.004 | *** | -0.060 | *** | -0.056 | *** |
| June 1-15 | 0.000 |  | 0.005 | *** | -0.073 | *** | -0.068 | *** |
| June 16-30 | 0.001 |  | 0.006 | *** | -0.082 | *** | -0.075 | *** |
| July 1-15 | 0.003 | * | 0.007 | *** | -0.091 | *** | -0.084 | *** |
| July 16-31 | 0.005 | *** | 0.008 | *** | -0.100 | *** | -0.093 | *** |
| Aug. 1-15 | 0.008 | *** | 0.008 | *** | -0.105 | *** | -0.097 | *** |
| Aug. 16-31 | 0.008 | *** | 0.008 | *** | -0.118 | *** | -0.109 | *** |
| Sept. 1-15 | 0.009 | *** | 0.009 | *** | -0.121 | *** | -0.112 | *** |
| Sept. 16-30 | 0.007 | ** | 0.008 | *** | -0.087 | *** | -0.079 | *** |
| Oct. 1-15 | 0.004 |  | 0.008 | * | -0.026 |  | -0.019 |  |
| Oct. 16-31 | -0.002 |  | 0.005 |  | 0.060 |  | 0.065 |  |
| Nov. 1-15 | -0.016 | *** | 0.002 |  | 0.071 |  | 0.074 |  |
| Nov. 16-30 | -0.029 | *** | -0.001 |  | 0.049 |  | 0.047 |  |
| Dec. 1-15 | -0.036 | *** | -0.004 |  | 0.050 |  | 0.046 |  |

*, **, *** denote statistical significance at the 10%, 5%, and 1% levels, respectively.

| MINOR | non-spatial | | Spatial Durbin Model | | | | | |
| --- | --- | --- | --- | --- | --- | --- | --- | --- |
|  | **Direct - OLS** | | **Direct** | | **Indirect** | | **Total** | |
|  | **Coeff.** | **Sig.** | **Coeff.** | **Sig.** | **Coeff.** | **Sig.** | **Coeff.** |  |
| May 1-16 | 0.007 | *** | 0.007 | *** | 0.039 | *** | 0.045 | *** |
| May 16-31 | 0.009 | *** | 0.009 | *** | 0.054 | *** | 0.064 | *** |
| June 1-15 | 0.011 | *** | 0.011 | *** | 0.067 | *** | 0.078 | *** |
| June 16-30 | 0.014 | *** | 0.014 | *** | 0.077 | *** | 0.091 | *** |
| July 1-15 | 0.018 | *** | 0.016 | *** | 0.096 | *** | 0.112 | *** |
| July 16-31 | 0.023 | *** | 0.018 | *** | 0.118 | *** | 0.136 | *** |
| Aug. 1-15 | 0.025 | *** | 0.020 | *** | 0.124 | *** | 0.144 | *** |
| Aug. 16-31 | 0.026 | *** | 0.020 | *** | 0.132 | *** | 0.152 | *** |
| Sept. 1-15 | 0.025 | *** | 0.019 | *** | 0.133 | *** | 0.153 | *** |
| Sept. 16-30 | 0.025 | *** | 0.019 | *** | 0.128 | *** | 0.147 | *** |
| Oct. 1-15 | 0.023 | *** | 0.019 | *** | 0.103 | *** | 0.122 | *** |
| Oct. 16-31 | 0.020 | *** | 0.020 | *** | 0.052 | ** | 0.072 | *** |
| Nov. 1-15 | 0.014 | *** | 0.020 | *** | -0.015 |  | 0.005 |  |
| Nov. 16-30 | 0.010 | *** | 0.022 | *** | -0.056 | * | -0.034 |  |
| Dec. 1-15 | 0.006 | * | 0.026 | *** | -0.089 | ** | -0.063 | * |

*, **, *** denote statistical significance at the 10%, 5%, and 1% levels, respectively.

| TRANSP | Non-spatial | | Spatial Durbin Model | | | | | |
| --- | --- | --- | --- | --- | --- | --- | --- | --- |
|  | **Direct - OLS** | | **Direct** | | **Indirect** | | **Total** | |
|  | **Coeff.** | **Sig.** | **Coeff.** | **Sig.** | **Coeff.** | **Sig.** | **Coeff.** | **Sig.** |
| May 1-16 | 0.001 |  | 0.001 |  | 0.032 | ** | 0.033 | ** |
| May 16-31 | 0.002 | ** | 0.002 | ** | 0.030 | * | 0.032 | ** |
| June 1-15 | 0.003 | *** | 0.003 | ** | 0.029 |  | 0.031 | * |
| June 16-30 | 0.004 | *** | 0.003 | *** | 0.038 | * | 0.041 | ** |
| July 1-15 | 0.004 | *** | 0.004 | *** | 0.044 | ** | 0.047 | ** |
| July 16-31 | 0.006 | *** | 0.005 | *** | 0.049 | ** | 0.054 | ** |
| Aug. 1-15 | 0.007 | *** | 0.006 | *** | 0.051 | * | 0.057 | ** |
| Aug. 16-31 | 0.008 | *** | 0.007 | *** | 0.037 |  | 0.044 |  |
| Sept. 1-15 | 0.009 | *** | 0.008 | *** | 0.012 |  | 0.020 |  |
| Sept. 16-30 | 0.009 | *** | 0.010 | *** | -0.061 | * | -0.051 |  |
| Oct. 1-15 | 0.010 | *** | 0.012 | *** | -0.165 | *** | -0.153 | *** |
| Oct. 16-31 | 0.012 | *** | 0.015 | *** | -0.314 | *** | -0.299 | *** |
| Nov. 1-15 | 0.016 | *** | 0.018 | *** | -0.427 | *** | -0.409 | *** |
| Nov. 16-30 | 0.021 | *** | 0.021 | *** | -0.451 | *** | -0.429 | *** |
| Dec. 1-15 | 0.027 | *** | 0.025 | *** | -0.474 | *** | -0.449 | *** |

*, **, *** denote statistical significance at the 10%, 5%, and 1% levels, respectively.

| EPIDEM | non-spatial | | Spatial Durbin Model | | | | | |
| --- | --- | --- | --- | --- | --- | --- | --- | --- |
|  | **Direct - OLS** | | **Direct** | | **Indirect** | | **Total** | |
|  | **Coeff.** | **Sig.** | **Coeff.** | **Sig.** | **Coeff.** | **Sig.** | **Coeff.** | **Sig.** |
| May 1-16 | 0.001 |  | 0.000 |  | 0.003 |  | 0.003 |  |
| May 16-31 | 0.001 |  | -0.001 |  | 0.017 |  | 0.016 |  |
| June 1-15 | 0.001 |  | -0.002 |  | 0.032 | * | 0.031 | * |
| June 16-30 | 0.002 | * | -0.002 |  | 0.041 | ** | 0.039 | ** |
| July 1-15 | 0.003 | *** | -0.002 |  | 0.061 | ** | 0.059 | ** |
| July 16-31 | 0.006 | *** | -0.002 |  | 0.089 | *** | 0.087 | *** |
| Aug. 1-15 | 0.007 | *** | -0.002 |  | 0.099 | *** | 0.096 | *** |
| Aug. 16-31 | 0.008 | *** | -0.004 | ** | 0.128 | *** | 0.124 | *** |
| Sept. 1-15 | 0.007 | *** | -0.007 | *** | 0.152 | *** | 0.145 | *** |
| Sept. 16-30 | 0.007 | *** | -0.007 | ** | 0.171 | *** | 0.164 | *** |
| Oct. 1-15 | 0.005 | ** | -0.007 | ** | 0.185 | *** | 0.178 | *** |
| Oct. 16-31 | 0.003 |  | -0.006 | * | 0.206 | *** | 0.200 | *** |
| Nov. 1-15 | -0.005 | * | -0.008 | ** | 0.203 | *** | 0.195 | *** |
| Nov. 16-30 | -0.009 | *** | -0.009 | *** | 0.220 | *** | 0.211 | *** |
| Dec. 1-15 | -0.011 | *** | -0.013 | *** | 0.278 | *** | 0.264 | *** |

*, **, *** denote statistical significance at the 10%, 5%, and 1% levels, respectively.

| HEALTH | non-spatial | | Spatial Durbin Model | | | | | | | | |
| --- | --- | --- | --- | --- | --- | --- | --- | --- | --- | --- | --- |
|  | **Direct - OLS** | | **Direct** | | | **Indirect** | | | **Total** | | |
|  | **Coeff.** | **Sig.** | | **Coeff.** | **Sig.** | | **Coeff.** | **Sig.** | | **Coeff.** | **Sig.** |
| May 1-16 | 0.000 |  | | 0.001 | * | | -0.031 | *** | | -0.030 | *** |
| May 16-31 | 0.001 |  | | 0.002 | ** | | -0.034 | *** | | -0.032 | *** |
| June 1-15 | 0.002 | ** | | 0.003 | ** | | -0.036 | *** | | -0.034 | *** |
| June 16-30 | 0.003 | *** | | 0.003 | *** | | -0.036 | *** | | -0.033 | ** |
| July 1-15 | 0.005 | *** | | 0.004 | *** | | -0.028 | * | | -0.024 | * |
| July 16-31 | 0.008 | *** | | 0.005 | *** | | -0.012 |  | | -0.007 |  |
| Aug. 1-15 | 0.011 | *** | | 0.005 | *** | | 0.015 |  | | 0.021 |  |
| Aug. 16-31 | 0.014 | *** | | 0.005 | *** | | 0.046 | ** | | 0.051 | *** |
| Sept. 1-15 | 0.016 | *** | | 0.004 | ** | | 0.073 | *** | | 0.078 | *** |
| Sept. 16-30 | 0.016 | *** | | 0.004 | ** | | 0.080 | *** | | 0.084 | *** |
| Oct. 1-15 | 0.014 | *** | | 0.004 | * | | 0.075 | *** | | 0.079 | *** |
| Oct. 16-31 | 0.011 | *** | | 0.004 |  | | 0.066 | * | | 0.070 | ** |
| Nov. 1-15 | 0.008 | *** | | 0.002 |  | | 0.101 | *** | | 0.103 | *** |
| Nov. 16-30 | 0.005 | * | | 0.003 |  | | 0.093 | ** | | 0.096 | ** |
| Dec. 1-15 | 0.004 |  | | 0.004 |  | | 0.062 |  | | 0.066 | * |

*, **, *** denote statistical significance at the 10%, 5%, and 1% levels, respectively.

| ENVIRON | non-spatial | | Spatial Durbin Model | | | | | | |
| --- | --- | --- | --- | --- | --- | --- | --- | --- | --- |
|  | **Direct - OLS** | | **Direct** | | **Indirect** | | | **Total** | |
|  | **Coeff.** | **Sig.** | **Coeff.** | **Sig.** | **Coeff.** | **Sig.** | **Coeff.** | | **Sig.** |
| May 1-16 | -0.002 | *** | -0.002 | *** | -0.020 | ** | -0.022 | | * |
| May 16-31 | -0.002 | * | -0.002 | ** | -0.026 | ** | -0.028 | | ** |
| June 1-15 | -0.002 | * | -0.002 | ** | -0.021 |  | -0.023 | | * |
| June 16-30 | -0.001 |  | -0.002 | ** | -0.014 |  | -0.016 | |  |
| July 1-15 | 0.000 |  | -0.002 | * | 0.000 |  | -0.002 | |  |
| July 16-31 | 0.001 |  | -0.002 |  | 0.024 |  | 0.023 | |  |
| Aug. 1-15 | 0.002 | * | -0.001 |  | 0.037 | * | 0.036 | | * |
| Aug. 16-31 | 0.004 | *** | -0.001 |  | 0.044 | * | 0.043 | | * |
| Sept. 1-15 | 0.005 | *** | 0.000 |  | 0.050 | ** | 0.049 | | ** |
| Sept. 16-30 | 0.008 | *** | 0.000 |  | 0.064 | ** | 0.064 | | ** |
| Oct. 1-15 | 0.011 | *** | 0.001 |  | 0.083 | *** | 0.084 | | *** |
| Oct. 16-31 | 0.015 | *** | 0.002 |  | 0.108 | *** | 0.110 | | *** |
| Nov. 1-15 | 0.024 | *** | 0.004 | * | 0.132 | *** | 0.137 | | *** |
| Nov. 16-30 | 0.028 | *** | 0.006 | ** | 0.101 | ** | 0.107 | | *** |
| Dec. 1-15 | 0.032 | *** | 0.009 | *** | 0.094 | ** | 0.103 | | ** |

*, **, *** denote statistical significance at the 10%, 5%, and 1% levels, respectively.

| POP DENSITY | non-spatial | | Spatial Durbin Model | | | | | | | |
| --- | --- | --- | --- | --- | --- | --- | --- | --- | --- | --- |
|  | **Direct - OLS** | | **Direct** | | | **Indirect** | | | **Total** | |
|  | **Coeff.** | **Sig.** | **Coeff.** | **Sig.** | **Coeff.** | | **Sig.** | **Coeff.** | | **Sig.** |
| May 1-16 | 0.005 | *** | 0.002 | ** | 0.025 | | *** | 0.027 | | *** |
| May 16-31 | 0.005 | *** | 0.001 |  | 0.030 | | *** | 0.031 | | *** |
| June 1-15 | 0.005 | *** | 0.000 |  | 0.031 | | *** | 0.031 | | *** |
| June 16-30 | 0.005 | *** | 0.001 |  | 0.027 | | *** | 0.028 | | *** |
| July 1-15 | 0.005 | *** | 0.003 | ** | 0.015 | | ** | 0.018 | | *** |
| July 16-31 | 0.005 | *** | 0.005 | *** | -0.001 | |  | 0.003 | |  |
| Aug. 1-15 | 0.005 | *** | 0.005 | * | -0.014 | |  | -0.010 | |  |
| Aug. 16-31 | 0.004 | *** | 0.004 | * | -0.031 | | *** | -0.027 | | *** |
| Sept. 1-15 | 0.003 | ** | 0.004 | * | -0.052 | | *** | -0.048 | | *** |
| Sept. 16-30 | -0.001 |  | 0.004 |  | -0.092 | | *** | -0.088 | | *** |
| Oct. 1-15 | -0.007 | *** | 0.003 |  | -0.143 | | *** | -0.140 | | *** |
| Oct. 16-31 | -0.017 | *** | 0.000 |  | -0.213 | | *** | -0.213 | | *** |
| Nov. 1-15 | -0.030 | *** | -0.002 |  | -0.302 | | *** | -0.304 | | *** |
| Nov. 16-30 | -0.040 | *** | -0.004 |  | -0.354 | | *** | -0.358 | | *** |
| Dec. 1-15 | -0.043 | *** | -0.010 | ** | -0.358 | | *** | -0.368 | | *** |

*, **, *** denote statistical significance at the 10%, 5%, and 1% levels, respectively.

**APPENDIX D. COVID-19 Community Vulnerability Index (CCVI)**


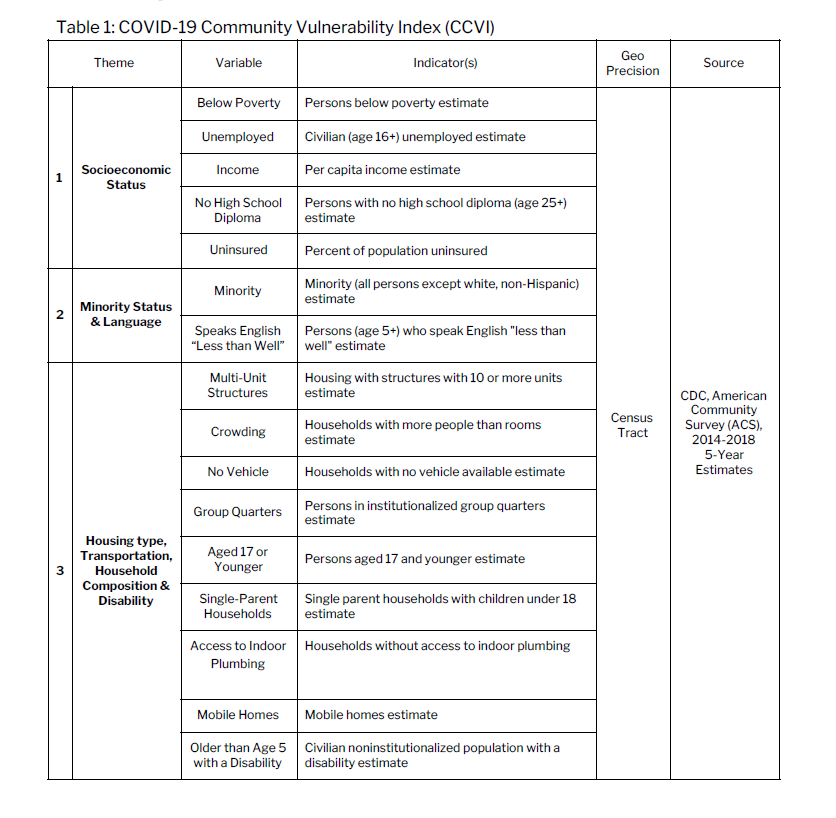


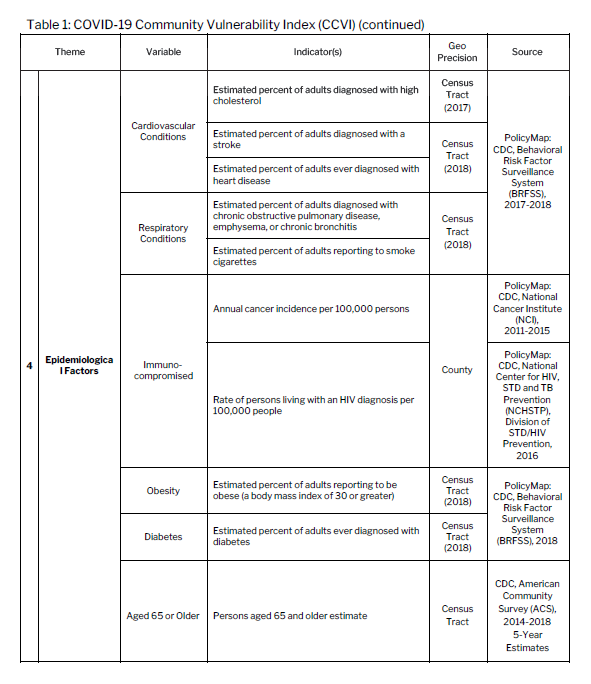


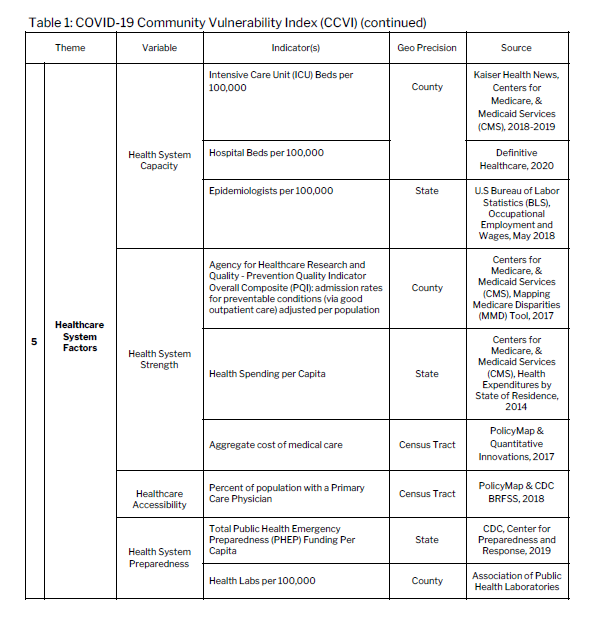


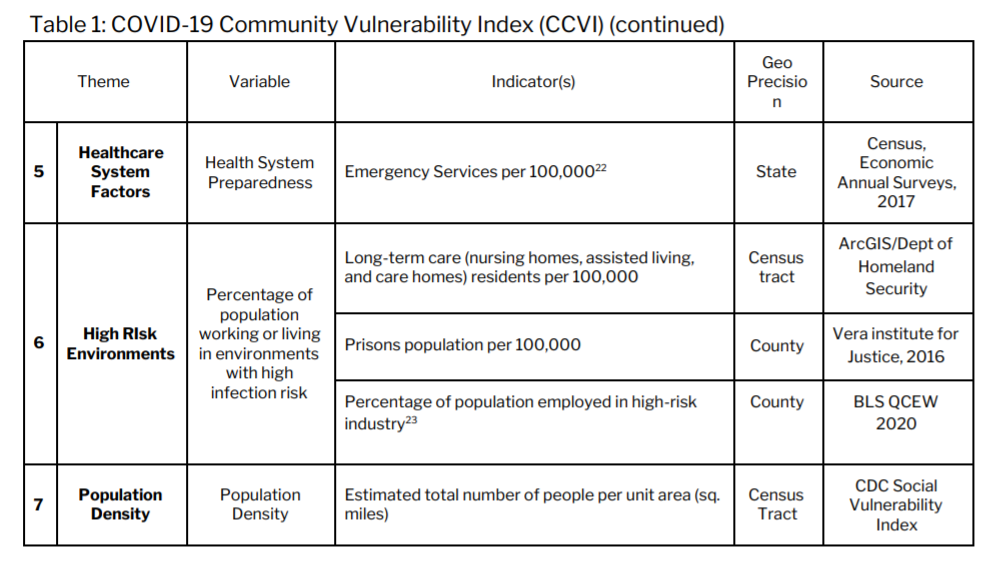


**(Source: Surgo Foundation)**
